# Supplementary material for: Women’s postpartum family planning use and intention in Ethiopia: Disparities in the agrarian and pastoral contexts from a community-based cross-sectional study
Source: PLOS Glob Public Health. 2026 Apr 27;6(4):e0005846. doi: 10.1371/journal.pgph.0005846 (PMC13119961; doi:10.1371/journal.pgph.0005846)
Supplement: Attachment — Submitted filename: Point by Point respons PLOS GPH R1.pdf [file pgph.0005846.s003.pdf]

Point by Point response

Manuscript

Title: Women's postpartum family planning use and intention in Ethiopia: Disparities in the agrarian and pastoral contexts from community-based cross-sectional study: ID: PGPH-D-25-04096
PLOS Global Public Health

Section	Comments	*Authors response*
Editor		
Journal Requirements:	1. Please provide a detailed online Financial Disclosure statement. This is published with the article. It must therefore be completed in full sentences and contain the exact wording you wish to be published. a) State the initials, alongside each funding source, of each author to receive each grant. For example: "This work was supported by the National Institutes of Health (######## to AM; ####### to CJ) and the National Science Foundation (###### to AM)."<br><br>b) State what role the funders took in the study. If the funders had no role in your study, please state: "The funders had no role in study design, data collection and analysis, decision to publish, or preparation of the manuscript."<br><br>For more information, please go to our submission guidelines: https://journals.plos.org/globalpublichealth/s/submission-guidelines#loc-financial-disclosure-statement<br><br>2. Please ensure that the funders and grant numbers match between the Financial Disclosure field and the Funding Information tab in your submission form. Note that the funders must be provided in the same order in both places as well.	*Thank you so much for the suggestion: we have included the financia statements* ***"Financial Declaration Statement:*** *This work was supported by the Gates Foundation [INV-002643 to MM and INV-037995 to DE]. The funder has no role in the study design, data collection, and analysis, decision to publish, or preparation of the manuscript. All these roles are the full responsibility of the all authors." See track change.*

	3. In the online submission form, you indicated that "All related data are presented fully within the paper, and available upon reasonable request to the lead author and the corresponding author.".<br><br>All PLOS journals now require all data underlying the findings described in their manuscript to be freely available to other researchers, either<br>a) In a public repository,<br>b) Within the manuscript itself, or<br>d) Uploaded as supplementary information.<br><br>This policy applies to all data except where public deposition would breach compliance with the protocol approved by your research ethics board. If your data cannot be made publicly available for ethical or legal reasons (e.g., public availability would compromise patient privacy), please explain your reasons by return email and your exemption request will be escalated to the editor for approval. Your exemption request will be handled independently and will not hold up the peer review process, but will need to be resolved should your manuscript be accepted for publication. One of the Editorial team will then be in touch if there are any issues. For further assistance, you may go to: http://journals.plos.org/globalpublichealth/s/data-availability	*The statement for data sharing included as : "**Availability of data and materials:** All related data are presented fully within the paper, and available upon reasonable request to the lead author and the corresponding author.*<br>*See track change "*
	4. Please provide separate main figure files in .tif or .eps format only and ensure that all files are under our size limit of 10MB. For more information about how to convert your figure files please see our guidelines: https://journals.plos.org/globalpublichealth/s/figures	*Okay figures shared in tif or eps. We coverted them in to *.tif; format*
	5. Please include a separate legend or caption for each figure in your main manuscript.	*Thanks, done. See trach change*
	6. Please upload a copy of "Supplement file 1", which you refer to in your text on page 6 and ensure that it has a legend listed in the manuscript before or after the references list. Or if the supplementary material is no longer to be included as part of the submission, please remove all reference to it within the text.	*Thanks, we have revised and we do not think that supplement file is necessary, and removed. See track change*
	7. If the reviewer comments include a recommendation to cite specific previously published works, please review and evaluate these publications to determine whether they are	*Thanks, done. See track change*

	relevant and should be cited. There is no requirement to cite these works unless the editor has indicated otherwise.	
	8. Please review your reference list to ensure that it is complete and correct. If you have cited papers that have been retracted, please include the rationale for doing so in the manuscript text, or remove these references and replace them with relevant current references. Any changes to the reference list should be mentioned in the rebuttal letter that accompanies your revised manuscript. If you need to cite a retracted article, indicate the article's retracted status in the References list and also include a citation and full reference for the retraction notice.	*Thanks, done. See track change*
Reviewer 1		
General	Reviewer #1: The authors looked at a very critical area of intervention in preventing maternal and child mortality and the study was done in hard to reach places to understand the use of PPFP which can be generalized further to similar areas. I have the following few general feedback which will help the manuscript to go out in a better shape.	*Thanks., comments addressed*
- Abstract/introduction:	- There is a description of Immediate PPFP- since the study looked at primarily on the extended period of PPFP and very limited descriptions on IPPFP, it is better to remove the word Immediate. In Ethiopia, Immediate Postpartum Family Planning (IPPFP) refers to providing family planning counseling and methods to women within 48 hours after childbirth, before they leave the health facility, to prevent unintended pregnancies and promote birth spacing, integrating services with early postpartum care for better maternal and child health outcomes.	*Thank you very much for the comments and the term indicated removed as per suggestion. See track change.*
	- Line-2 in the introduction- the word CONTROL is very controversial and the reference document you put gives a better wording on the use of contraception. "Family Planning allows people to attain their desired number of children and determine the spacing of pregnancies".	*Thanks, corrected as "attain the desired number of children' see track change*
	- Line 24 in the introduction. beyond health care decision, the quality of health care is a very critical issue which is not described. Since most delivering mothers are discharged without a contraceptive method, one should ask on the quality of post-partum care. This might be a useful reference to look at it- Silesh M, Demisse TL, Taye BT, Moltot T, Chekole MS, Wogie G, Kasahun F, Adanew S. Immediate postpartum family planning utilization and its associated factors among postpartum women in Ethiopia: a systematic review and meta-analysis. Front Glob	*Thank you so much for the suggestion and quality of service add and cited accordingly, see trach change*

	Women's Health. 2023 Aug 22;4:1095804. doi: 10.3389/fgwh.2023.1095804. PMID: 37674902; PMCID: PMC10478094.	
- Methods:	- Line-2 in the project description- Figure-2 is a theory of change not PPFP service distribution. Please check it again.	*Thanks, the ToC has PPFP component. Intervention or lifesaving package component*
- Results:	- Line-19 under the results section- Post Partum uptake- 2.5% of the participants has immediate PPFP within 48 hours and it is good to include on how many of them had facility births.	*Thanks, though facility birth is very low, majority of women who gave birth at health facility used ppfp (n=535/762).*
	- Table-3 title needs more clarity- Since preferred choice is not always actual use and you need to revise the title to represent actual use. The table represents the actual contraceptive use.	*Thanks, this is women's self-reported data and we respectfully accepted their self-reported data*
	- Under results line 6-8 there seems a calculation error- with 762 users of which 752 use their preferred choice which should be 98.7% and the remaining 10 in like 1.3% without their preferred choice. Please correct the error.	*Thanks, corrected the calculation this is women's self-reported data and we respectfully accepted their self-reported data. See track change*
	- On table-4 on reasons of not using PPFP, is the religious prohibition a real one or an individual perception? Since a large percentage of the respondents fall under that category it is good to describe that in both the results and discussion section.	*Thanks, included some as per the suggestion. Descriptions are located above the table using text along with citation of Table 4*
- Discussion:	- The are a number of findings in the study and I expect more to be described in the discussion section. As of now it is limited with three paragraphs with primarily comparing with other studies and very limited justification on some of the results.<br>As an example, I don't see any description on the reason of having almost a 2.5% use of Immediate PPFP within 48 hours, while almost close to 32% delivered at facilities and most	*Thanks, a new paragraph is added to accommodate the given comments, and enhance readability of the*

	left without contraception. It is good to look at some of the justification which can be used for a call to action for anyone using the results of such studies.	*manuscript. see track change*
	In few areas there is a need for word editing as an example Recall bias is written as Recall Basis, pastoral setting as Pastoral petting.	*Thanks, made editings and address the concern as much possible*
	Reviewer 2 -	
- Introduction:	- In the statemen of the problem section, the problem reflected a real researchable gap or challenge. But there are some confusing sentences to be considered like line numbers 32 and 33: 32 is stated as: "contexts has not been recognized but is well explored and comprehensively documented through previous studies.’ These sentences seem to contradict one another and have clarity and logic problems. As written, it seems the wo ideas cancel each other out.	*Thanks for the comment. And corrected as per the suggestion to improve readability. See track change*
- Methods:	- The methods section starts by stating the ethical issues, which is not the main focus of the section. It has to come at the end of the section. It has to follow this sequence: Study setting, study design, population, sample and sampling techniques, method of data analysis, data presentation, eligibility criteria, and ethical clearance.	*Thanks, the first section og the method is the ethical consideration as per the journal guideline to authors. Thanks*
	- In the "Study design and participants" sub-section, there is a redundancy in numbers 6 & 7 and numbers 10-12.	*Thanks, double checked*
