# Supplementary material for: Women’s postpartum family planning use and intention in Ethiopia: Disparities in the agrarian and pastoral contexts from a community-based cross-sectional study
Source: PLOS Glob Public Health. 2026 Apr 27;6(4):e0005846. doi: 10.1371/journal.pgph.0005846 (PMC13119961; doi:10.1371/journal.pgph.0005846)
Supplement: Attachment — Submitted filename: Point by point response R21.pdf [file pgph.0005846.s004.pdf]

PGPH-D-25-04096R2

Women’s postpartum family planning use and intention in Ethiopia: Disparities in the agrarian and pastoral contexts from community-based cross-sectional study

Comment

1. In the online submission form, you indicated that "All related data are presented fully within the paper, and available upon reasonable request to the lead author and the corresponding author."

Response: included in the manuscript and online submission

Thanks
